# Autoimmune glial fibrillary acidic protein astrocytosis mimicking tuberculous meningitis: a retrospective study

**DOI:** 10.1007/s00415-023-11818-8

**Published:** 2023-06-20

**Authors:** Yingfang Liang, Gangqi Wang, Bixun Li, Guoliang Li, Hao Zeng

**Affiliations:** 1 Department of Comprehensive Internal Medicine, The Affiliated Tumor Hospital of Guangxi Medical University, 71#Hedi Rd, Nanning, GuangXi China; 2 Department of Neurology, Xiangya Hospital, Central South University, 87#Xiangya Rd, Changsha, Hunan China; 3 Department of Spine and Osteopathy Surgery, The First Affiliated Hospital of Guangxi Medical University, 6#Shuangyong Rd, Nanning, GuangXi China

**Keywords:** Glial fibrillary acidic protein, Astrocytosis, Cerebrospinal fluid, Tuberculous meningitis, Lymphocytosis

## Abstract

**Background:**

This study aimed to summarize the clinical features of Autoimmune Glial Fibrillary Acidic Protein Astrocytosis mimicking tuberculosis meningitis to improve clinicians’ understanding of this disease.

**Methods:**

We retrospectively analyzed the clinical manifestations, cerebrospinal fluid results, and imaging data of five patients with Autoimmune Glial Fibrillary Acidic Protein Astrocytosis mimicking tuberculous meningitis who were admitted to Xiangya Hospital Central South University between October 2021 and July 2022.

**Results:**

Five patients were aged 31–59 years, with a male-to-female ratio of 4:1. Among the cases reviewed, four had a history of prodromal infections manifesting as fever and headache. One patient developed limb weakness and numbness with clinical manifestations of meningitis, meningoencephalitis, encephalomyelitis, or meningomyelitis. Cerebrospinal fluid analysis revealed an increased cell count in five cases, with a lymphocyte majority. All five cases had a CSF protein level > 1.0 g/L, CSF/blood glucose ratio < 0.5, and two patients had CSF glucose < 2.2 mmol/L. Decreased CSF chloride was observed in three cases, while increased ADA was observed in one case. Both serum and cerebrospinal fluid were positive for anti-GFAP antibodies in three cases, while in two cases, only CSF was positive for anti-GFAP antibodies. Additionally, hyponatremia and hypochloremia were observed in three cases. No tumors were detected in any of the five patients during tumor screening, and all five cases had a good prognosis following immunotherapy.

**Conclusion:**

Anti-GFAP antibody testing should be routinely performed in patients with suspected tuberculosis meningitis to avoid misdiagnosis.

## Introduction

Autoimmune glial fibrillary acidic protein astrocytopathy (A-GFAP-A) is a group of autoimmune neurological diseases [1, 2] affecting the brain, meninges, spinal cord, and optic nerve. GFAP is an intermediate filament protein essential for the cytoskeletal structure of adult astrocytes. In 2016, FANG et al. [1] reported that GFAP-IgG in the cerebrospinal fluid (CSF) and/or serum by cell-based testing could be a potential biomarker for diagnosing this disease. Clinical symptoms include fever, headache, brain and meningeal abnormalities, imaging and cerebrospinal fluid changes, lack of specificity, and susceptibility to misdiagnosis. Especially when the cerebrospinal fluid depicts tuberculous lesions, it is easily confused with tuberculous meningitis (TBM) [3–9]. This study retrospectively examined the clinical features of A-GFAP-A in patients with tuberculous meningitis to improve understanding and diagnostic efficacy.

## Materials and methods

### Research objects

This research retrospectively analyzed five patients with A-GFAP-A and clinical features of tuberculous meningitis who were admitted to the Neurology Department of Xiangya Hospital of Central South University between October 2021 and July 2022. The patients satisfied the following inclusion criteria[1]: (1) The clinical manifestations were similar to those of encephalitis, meningitis, myelitis, encephalomyelitis, or meningomyelitis; (2) all patients underwent serum and/or CSF testing, and all of them tested positive for CSF GFPA antibodies through cell-based assay (CBA).

### Research methodology

#### Data collection

The collected and analyzed clinical data included age, gender, first symptoms, main clinical manifestations, laboratory tests, imaging tests, treatment plans, and follow-up prognoses by phone.

#### Cell‑based assay

This study used a cell-based assay (CBA) method because of its high specificity and sensitivity. CBA is a cell transfection-based indirect immunofluorescence assay. In this assay, GFAP genes are transfected into mammalian cells to express GFAP antigens in the mammalian cytoplasm. Green fluorescent proteins (GFP) were also expressed in the transfection as an internal reference for detection. The transfected cells were then fixed onto 96-well microplates to make antigen plates. GFAP antibodies in human serum, plasma, and cerebrospinal fluid samples were detected semi-quantitatively using indirect immunofluorescence.

The autoantibodies were detected using the CBA method. Briefly, the serum to be tested was diluted 1:10, or the original fluid of cerebrospinal fluid was used to incubate the patch of cells at room temperature for 60 min. The patch of cells was then treated with detergent three times, for five min each. The FITC-labeled goat anti-human IgG secondary antibody was then diluted 1:50 and incubated in the cell patch for 30 min at room temperature. The patch of cells was then cleansed, sealed, and observed under a fluorescence microscope. The fluorescence signal was significantly higher than the background signal, allowing the serum in the cell membrane to be identified as the positive serum of autoantibodies. When testing the titer of the positive serum, the serum or cerebrospinal fluid was diluted in a gradient, and the method described above was followed. The titer value of the positive serum was determined as the highest dilution multiple of the detectable positive signal.

#### Prognostic assessment

The prognosis was assessed using the modified Rankin Scale (mRS). An mRS score of 0 ~ 2 was considered a good prognosis, whereas a score of > 2 was considered poor.

## Results

### Clinical features

The clinical characteristics are summarized in Table 1. The reviewed cases included four males and one female. All patients reported subacute disease with clinical manifestations consistent with meningoencephalitis, meningitis, encephalomyelitis, or meningomyelitis. In four cases, acute meningeal symptoms manifested as neck stiffness. Prodromal infections, fever, and headache were observed in four patients, respectively. There were signs of cognitive function decline in three of the reviewed cases. Urinary and fecal passage difficulties were observed in two patients. One patient exhibited vomiting, slurred speech, limb tremors, unsteady walking, lower extremities weakness, limb weakness and numbness, autonomic system dysfunction, and intestinal paralysis.

**Table 1:** Clinical features of the patients with A-GFAP-A

Cases no	Age (years old)	Gender	Disease course	Initial symptoms	Main clinical symptoms	Clinical manifestations in the acute stage
1	59	Male	0.5 month	Fever and headache	Limb tremors and cognitive function decline	Meningoencephalitis
2	31	Male	10 days	Fever and headache		Meningitis
3	59	Male	20 days	Fever and headache	Unsteady walking, slurred speech, and cognitive function decline	Meningoencephalitis
4	53	Female	20 days	Fever and headache	Vomiting, urinary and bowel disorders, and numbness and weakness in both lower limbs	Meningomyelitis
5	59	Male	2 months	Weakness and numbness of limbs	Urinary and bowel disorders and cognitive function decline	Meningoencephalomyelitis

### Lab tests

1. Cerebrospinal fluid (CSF) findings: Three patients had normal cerebrospinal fluid pressure, while the other two were presented with elevated pressure (reference range: 80–180 mmHg). High white blood cells count (reference range: 0–10 × 10^6^/L), predominantly lymphocytes, was observed in five patients. Elevated CSF protein levels with values > 1.0 g/L (reference range: 0.15–0.45 g/L) were observed in all five cases. The CSF/blood glucose ratio < 0.5 was observed in all five cases, and in three cases, the absolute CSF glucose level (reference range: 2.5–4.4 mmol/L) was reduced. All five patients had reduced CSF chloride levels (reference range: 120–130 mmol/L), while one patient had elevated adenosine dehydrogenase (ADA) (reference range: 0–4 U/L). CSF immunoglobulin studies revealed that coxsackie virus IgM and human herpesvirus IgM were positive in one case, respectively. All five patients were negative for CSF mycobacterium tuberculosis smears, CSF mycobacterium tuberculosis cultures, CSF mycobacterium tuberculosis antibodies, CSF bacteriological culture, and CSF fungal culture (Table 2).
2. Blood serum tests: Among the patients included, three had hyponatremia (reference range: 137–147 mmol/L) and hypochlorhydria (reference range: 99–110 mmol/L, Table 2).
3. Antibody test: Positive GFAP serum and CSF antibodies were observed in three cases, while in the remaining two cases, it was only positive in CSF. Weakly positive anti-amphiphysin, anti-SSA, and anti-Ro-52 antibodies were detected in the pooled serum of one patient. Both serum and CSF were negative for MOG and AQP4 antibodies in all five cases.
4. Other tests: The tuberculosis diagnostic tests, i.e., T-spot and PPD skin test, were negative for all patients. Pulmonary CT, HIV, serum Treponema pallidum hemagglutination test (TPHA), serum tumor marker, and thyroid function tests did not reveal any abnormalities.

**Table2:** Lab test results of the patients with the A-GFAP-A

Case no	Blood sodium	Blood chloride	Blood glucose			Cerebrospinal	fluid			GFAP antibody
			Pressure	Nucleated cell count	Protein	Glucose	Chloride	ADA	Serum cerebrospinal fluid	
	mmol/L	mmol/L	mmol/L	mmH20	/ul	g/L	mmol/L	mmol/L	u/l	
1	132.0	92.0	7.46	90	130(90%)	1.55	3.07	115.5	3.1	Negative 1:100
				85	120(90%)	2.24	2.88	116.6	3.0	
	134.0	96.5	6.22	90	106(90%)	1.17	2.93	119.7	2.3	All data were measured before the treatment
2	129.1	93.4	6.79	180	60(70%)	1.09	2.99	116.0	3.7	1:32 1:100
	130.9	93.9	7.7	170	80(98%)	1.10	3.67	118.2	2.8	All data were measured before the treatment
	138.2	99.1	5.32	168	40(95%)	0.45	3.96	116.6	1.3	All data were measured during the re-examination in Day 10 after the treatment
3	132.4	95.7	8.56	85	48(90%)	1.27	1.80	116.0	3.7	1:32 1:10
	131.2	97.1	8.66	80	52(90%)	1.21	2.10	116.7	3.4	All data were measured before the treatment
	139.1	98.4	4.8	100	40(90%)	0.71	3.82	118.8	1.7	All data were measured during the re-examination in Day 9 after the treatment
4				240	180(90%)	1.05	2.11	100.8	13	Negative 1:1
	138.0	102.4	12.66	150	180(90%)	1.65	3.57	124.4	3.2	All data were measured before the treatment
5				240	20(90%)	1.18	2.80	115.0	7.6	1:100 1:32
	137.0	101.7	5.7	160	22(90%)	1.15	2.38	118.8	3.0	All data were measured before the treatment

Note: () is the percentage of lymphocytes over nucleated cells

### MRI findings

**Fig. 1:**
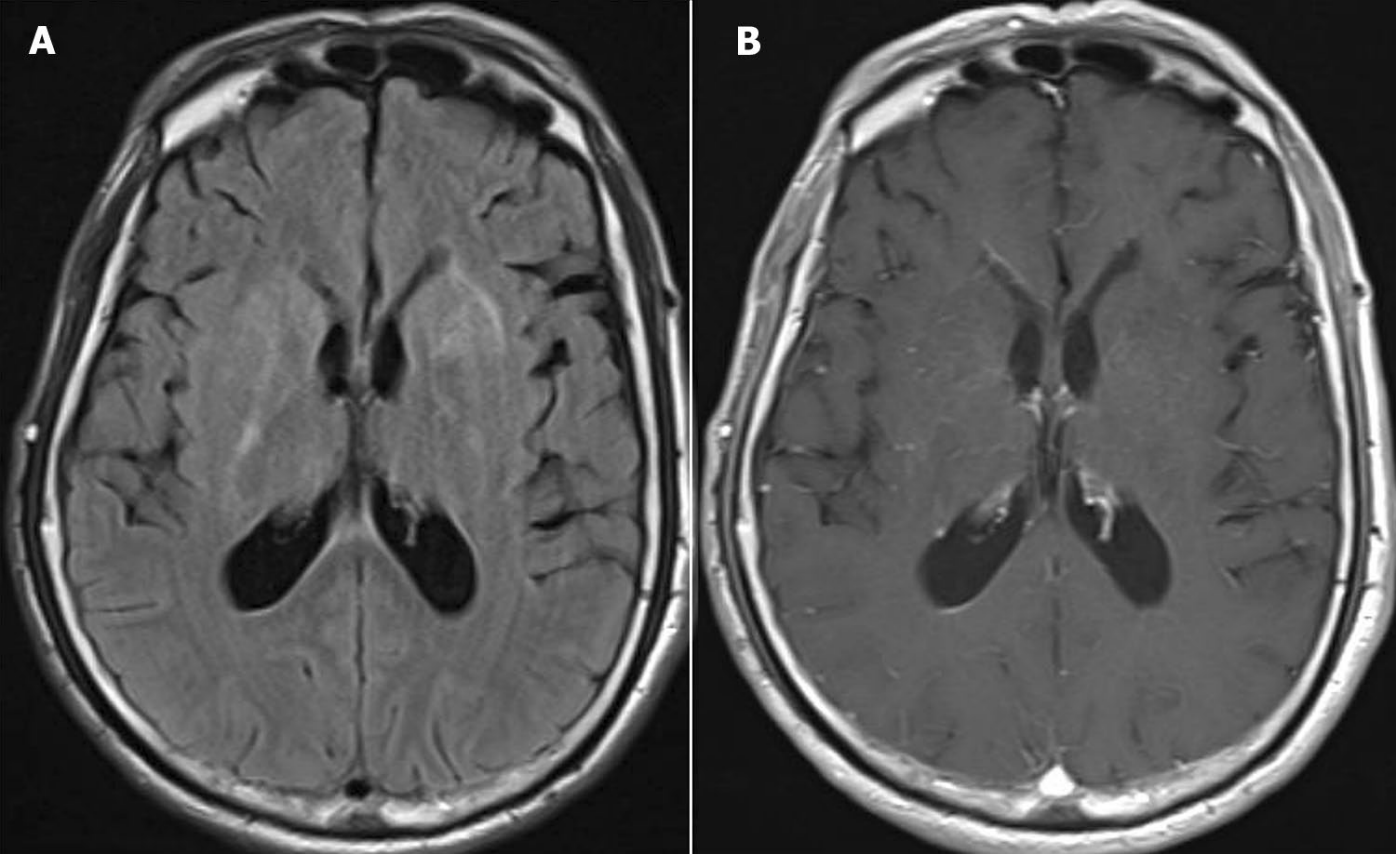
Brain MRI showing bilateral basal ganglia with the Flair image revealing high signals (**A**) without enhancement (**B**)

**Fig. 2:**
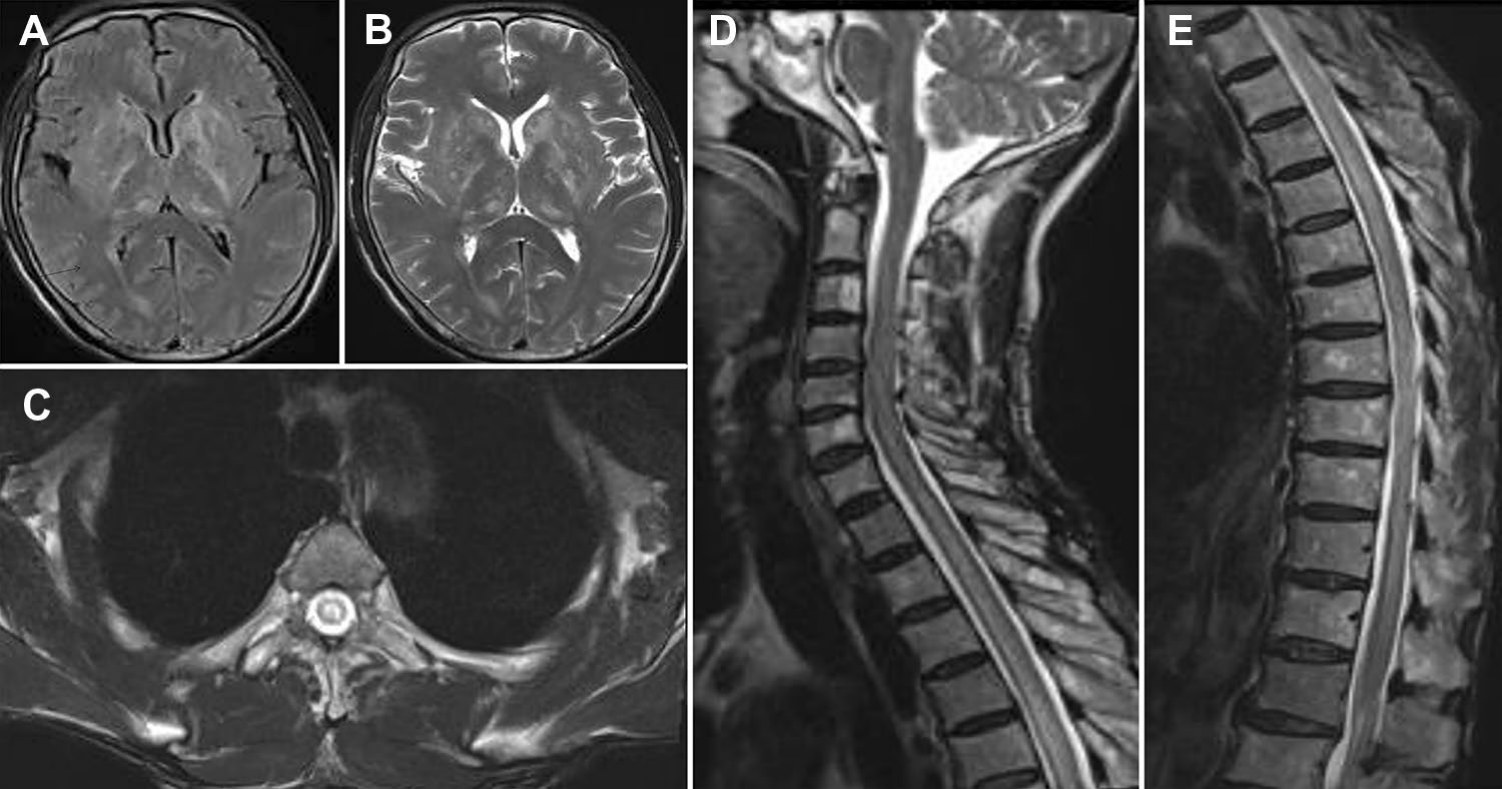
Brain MRI showing T2 patchy signal abnormalities in the bilateral basal ganglia, thalamus, and right corpus callosum, with the Flair image revealing high signals. (**A**, **B**). A slightly thickened spinal cord in the cervicothoracic segment, in which the slightly long T2 signals could be seen (**C**, **D**). These signals were mainly in the central part of the spinal cord (**E**)

**Fig. 3:**
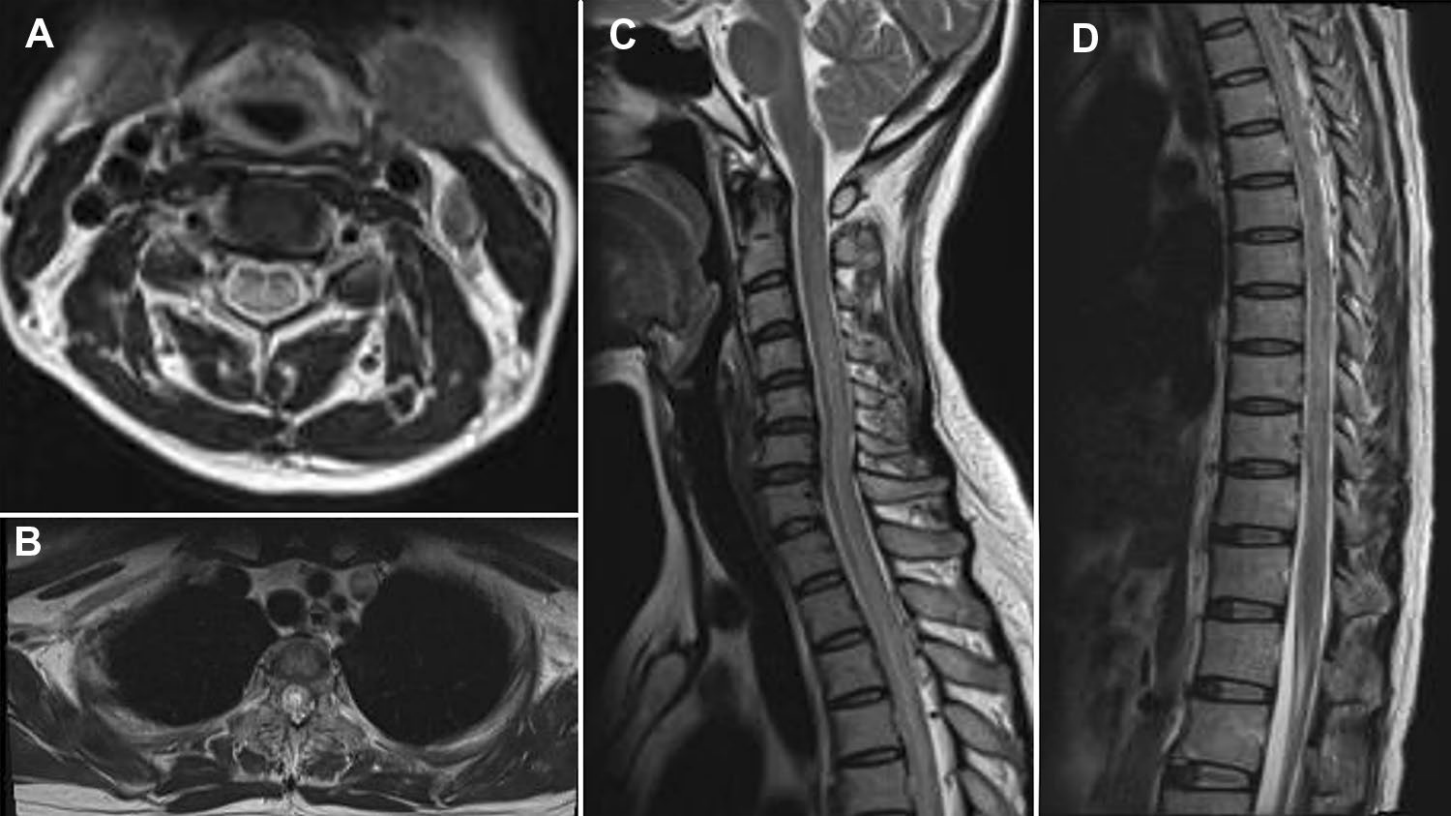
Spinal cord MRI showing strip and sheet-shaped long T2 signals in the horizontal spinal cord from the 2nd cervical vertebra to the 1st lumbar vertebrae, mainly involving the central part of the spinal cord (**A**, **B**, **C**, and **D**)

The results of MRI findings are displayed in Table 3. All five patients received Brain MRI scans for enhancement but did not exhibit any enhancement. Among them, the scan of case 1 revealed bilateral basal ganglia and left corpus callosum abnormalities on both T1- and T2-weighted images, with high signals being revealed on the Flair image (Fig. 1). Case 5 revealed multiple T1 and T2 patchy signal abnormalities in the bilateral basal ganglia, thalamus, and right corpus callosum (Fig. 2), whereas the other three cases demonstrated no anomalies. Spinal MRI scan enhancement was performed on three patients. Among them, case 4 displayed strip and sheet-shaped equal T1 and long T2 signals in the horizontal spinal cord from the 2nd cervical vertebra to the 1st lumbar vertebrae without enhancement, primarily affecting the central part of the spinal cord (Fig. 3). Case 5 revealed a slightly thickened spinal cord in the cervicothoracic segment, in which the slightly long T1 and T2 signals could be observed (Fig. 2). These signals were mainly located in the central part of the spinal cord. After enhancement, the spot and sheet-shaped signals were enhanced. No abnormal occurrences were observed in case 1.

**Table 3:** MRI test results of the patients with A-GFAP-A

Case No	Head	Spinal cord
1	Abnormal signals in the bilateral basal ganglia and left corpus callosum, showing long T1 and long T2 signals without enhancement	Normal
2	Normal	No test
3	Normal	No test
4	Normal	Strip and sheet-shaped equal T1 and long T2 signals are displayed in the horizontal spinal cord from the 2nd cervical vertebra to the 1st lumbar vertebrae without enhancement, mainly involving the central part of the spinal cord
5	Multiple long T1 and long T2 patchy signals displayed are in the bilateral basal ganglia, thalamus, and right side of the corpus callosum without enhancement	A slightly thickened spinal cord in the cervicothoracic segment, in which the slightly long T1 and T2 signals could be seen. These signals were mainly in the central part of the spinal cord. After enhancement, the spot and sheet-shaped signals were enhanced

### Electrophysiological test

Video EEG tests were performed in three cases, one of which revealed diffuse slow waves.

### Treatment and outcome

In the acute stage, all patients reviewed in this study received either glucocorticoid shock therapy or combination therapy with plasma exchange, and all of them responded well to treatment, as evidenced by the reduced mRS scores. The follow-up period was from the 2nd to the 8th month, with an average follow-up time of four months. During the most recent follow-up, the mRS scores of four cases were less than 2, while the mRS score of the remaining cases was 3, but without recurrence. There was no evidence of tumor recurrence in any reviewed patients during the follow-up period.

## Discussion

In this study, all five patients reviewed had a subacute disease, with fever and headache being the first symptoms for four patients. The primary clinical syndromes were meningitis, meningoencephalitis, encephalomyelitis, or meningomyelitis. Pulmonary CT, T-SPOT, PPD skin test, CSF mycobacterium tuberculosis cultures, CSF bacteriological culture, CSF fungal culture, HIV, TPHA, and serum tumor marker were negative for all patients. Their cerebrospinal fluid analysis revealed increased cell counts, with predomination of lymphocytes, elevated CSF proteins > 1.0 g/L, and CSF/blood glucose ratio < 0.5. All cases tested positive for GFAP antibody in CSF, and their response to corticosteroid therapy was excellent, confirming the diagnosis of A-GFAP-A. The clinical symptoms, cranial imaging changes, and other CSF anomalies of the five A-GFAP-A patients reported in this study were similar to those of tuberculous meningitis (TBM). According to previous studies, the misdiagnosis rate ranges between 4.5% and 35.7% [3, 4]. The presence of mycobacterium tuberculosis in cerebrospinal fluid is required for a definitive diagnosis of TBM. However, the pathological identification of mycobacterium sp. in CSF is clinically challenging; consequently, it is primarily diagnosed based on clinical symptoms, cranial imaging changes, other CSF anomalies, and response to diagnostic antituberculosis therapy, particularly in TB-endemic regions.

In this study, all five patients underwent brain MRI scans enhancement and demonstrated no enhancement. The brain MRI images of two patients depicted abnormal T2-weighted and FLAIR imaging of hyperintensity signals in the bilateral basal ganglia. Moreover, the brain MRI images of one patient exhibited abnormal T2-weighted and FLAIR imaging of hyperintensity signals in the bilateral thalamus lesion. Although previous studies indicate that linear perivascular radial gadolinium enhancement in the brain is a characteristic manifestation of the disease [2, 4, 10, 11], it has been suggested that radial perivascular emphasis is not necessarily associated with GFAP antibodies [12]. In this study, none of the five patients demonstrated linear perivascular radial gadolinium enhancement of the brain. Previous studies have reported that abnormal high-head MR signals in A-GFAP-A and TBM patients can involve the basal ganglia and thalamus [2–5, 10, 13–15]. Kimura et al. [4] even suggested that the bilateral thalamus abnormal hyperintense signal was the defining feature of A-GFAP-A. Zhuang et al. [10] depicted that patients with A-GFAP-A who had a high signal in the bilateral thalamus, basal ganglia region, and periventricular white matter accumulated inflammatory cells, antigens, and antibodies in the perivascular and V-R spaces. In cases with typical brain MRI findings (perivascular radial gadolinium enhancement), clinicians may easily suspect A-GFAP-A. However, in patients without a typical brain MRI finding, T2WI/FLAIR lesions involving bilateral basal ganglia and thalamus may make it difficult to differentiate between these two diseases. The rapid disappearance of brain MRI abnormalities in A-GFAP-A after corticosteroid administration may be a distinctive feature [16]. TBM can only resolve after anti-TB treatment. Therefore, the treatment efficacy may be a differentiating factor.

In this study, the similarities and differences between A-GFAP-A and TBM were primarily discussed regarding cerebrospinal fluid changes to improve the understanding of clinicians about the disease. In this study, five cases had elevated CSF cell counts, with lymphocytes predominating. Elevated CSF proteins > 1.0 g/L were also observed in all five cases. Elevated CSF lymphocytes, protein is more common in TBM; autoimmune disease is uncommon. Studies have revealed that the cerebrospinal fluid of A-GFAP-A patients is predominantly characterized by inflammatory changes and that CSF lymphocytes and proteins can be increased, with some patients exhibiting CSF protein > 1.0 g/L [2, 4–6, 8–10]. A CSF protein threshold of > 1.0 g/L (100 mg/dL) differentiated between cases of TBM, bacterial meningitis[17], and viral meningitis [18]. Solomons et al. [19] demonstrated that CSF protein > 1.0 g/L has a sensitivity of 78% and specificity of 94% for diagnosing TBM. In this study, CSF protein levels in all five cases were > 1.0 g/L, indicating that elevated CSF protein levels are not unique to TBM diagnosis. According to this study, a mild increase in CSF WBC in patients with A-GFAP-A was usually mismatched with a significant increase in CSF protein. CSF WBCs and CSF protein were concurrently significantly increased in patients with TBM. This may be one of the distinguishing factors between the two.

In this study, the CSF/ blood glucose ratio in all five cases was < 0.5, and two patients had CSF glucose < 2.2 mmol/L. The patient demonstrated decreased CSF glucose but normal serum glucose, which is hardly seen in autoimmune diseases. Studies have shown that the decrease in CSF glucose in A-GFP-A patients is only 15–18% [2, 13]. Reduced CSF glucose has been reported to be more prevalent in TBM. Solomons et al. [19] reported that a CSF glucose concentration of < 2.2 mmol/L diagnosed TBM with a sensitivity of 68% and a specificity of 96%. The sensitivity using a CSF to serum glucose ratio of < 0.5 was 90%. In a study by Jipa et al. [20], 90.3% of their TBM patients had CSF/blood glucose ratios < 0.5. Since CSF glucose concentration is highly dependent on blood glucose concentration, the CSF/ blood glucose ratio is more significant in the differential diagnosis to exclude blood factors. This study observed a similar change, which prompted the specificity and sensitivity not to be unique to TBM, necessitating the use of A-GFAP-A as a possible differential diagnosis. The decrease of CSF glucose in TBM patients may be due to the release of glycolytic enzymes from the brain and the consumption of glucose, whereas the cause of the decrease of glucose in A-GFAP-A patients remains unknown.

In this study, one patient experienced a transient increase in CSF adenosine deaminase (ADA) activity, which recovered spontaneously. Kimura et al. [4] reported that 71.4% of A-GFAP-A patients had a transient increase in ADA level within the first month of onset, which they regarded as a unique feature of the CSF of early A-GFAP-A patients. Nakamura et al. [21] reported elevated ADA levels in the cerebrospinal fluid without mycobacterium tuberculosis infection in support of A-GFAP-A diagnosis. ADA is essential in the growth and differentiation of lymphocytes and macrophages. Kimura et al. [4] reported that elevated ADA levels might be associated with immunological pathogenesis during the early stage of A-GFAP-A. Changes in ADA levels in the CSF of one patient in this study were similar to those described in the literature, with a transient increase in ADA followed by spontaneous recovery. This spontaneous recovery contrasts with the elevated CSF ADA found in TBM patients who only recover after antituberculosis therapy.

We reported five cases of A-GFAP-A with a clinical presentation and CSF profile resembling TB meningitis. We consider A-GFAP-A an important differential diagnosis, particularly in TB-endemic regions. In clinical practice, CSF GFAP-igg should be considered for early evaluation in patients with suspected tuberculous meningoencephalitis and atypical characteristics, and diagnostic antituberculosis should be administered with caution.

## Data Availability

The corresponding author has full access to all data and has the right to publish any and all data separate and apart from any sponsor.
